# Mapping the climate niches of forest insects and diseases in Canada under current and future climate

**DOI:** 10.1038/s41598-025-24833-8

**Published:** 2025-11-20

**Authors:** John H. Pedlar, Daniel W. McKenney, Glenn Lawrence

**Affiliations:** Natural Resources Canada, Canadian Forest Service – Great Lakes Forestry Centre, 1219 Queen Street East, Sault Ste. Marie, ON Canada

**Keywords:** Ecology, Ecology, Environmental sciences

## Abstract

Insects and diseases are important disturbance agents in Canadian forests and there is concern that their impacts will intensify under climate change. Here we report on an effort to model and map the climate niches of more than 4000 forest insect and fungus species in Canada – including high-profile pest species that are already, or may soon become, established in the country. This work employs occurrence data from historical, national-scale forest insect and disease surveys, several research collections, and the Global Biodiversity Information Facility (GBIF). We further employ national forest inventory products (gridded maps) to summarize forest host volumes at risk of infestation by selected insect and disease species. Maps of current and projected climate suitability have been made publicly available via a web application (https://cfs.cloud.nrcan.gc.ca/bmfid/), which allows the products to be explored and downloaded. We demonstrate use of the products through examples, including brown spruce longhorn beetle (*Tetropium fuscum*), southern pine beetle (*Dendroctonus frontalis*), oak wilt (*Bretziella fagacearum*), and map overlays that show hotspots for bark beetles under current and projected climate. We hope this tool will help pest managers to better understand how these species may respond to projected climate change over the course of the current century.

**Supplementary Information:**

The online version contains supplementary material available at 10.1038/s41598-025-24833-8.

## Introduction

Insects and diseases represent major disturbance vectors in Canadian forests^1^ where they are responsible for damaging, on average, more than 15 million hectares of forest annually^2^. Knowledge of current and future distributions of these organisms can assist forest managers and policy makers in anticipating and responding to potential pest threats^3–5^. Climate change provides an important motivation for such work, given the well-established ties between insect populations and climate^6–8^ and the prospects for rapid climate change in Canada during this century^9^. Here, we use the term ‘pest’ to refer to species that may negatively impact some aspects of forest health or condition, and the term ‘invasive’ to indicate a subset of pests that are not native to Canada and have shown the ability to spread rapidly and cause significant damage to forest resources.

Over the past several decades there has been a rapid development in methods available for modelling species distributions^10,11^. Bioclim^12,13^ – one of the earliest approaches – provides basic statistical summaries (i.e., min, max, mean, and percentiles) for a suite of climate variables at known species occurrence locations. Alternative approaches, which have generally increased in complexity through time, include regression methods^14^, genetic algorithms^15^, classification and regression trees^16^, and hierarchical Bayesian methods^17,18^. MaxEnt^19^ is a widely used machine learning method that has consistently performed well in comparison to other approaches^10,20,21^. In the current study, we employ two modelling approaches to generate potential distributions in relation to climate, which are hereafter referred to as climate niche or climate habitat models.

Modelling the climate niches of insects and diseases requires a variety of data inputs, many of which are increasingly and freely available due to the ongoing movement toward open data sharing. For example, georeferenced occurrence data for many forest insect and disease species are available through the Global Biodiversity Information Facility (GBIF)^22^. Furthermore, spatial datasets of Canadian forest attributes (i.e., information on insect and disease host distribution and abundance) have recently been developed and made available^23,24^. Finally, spatial climate data has been made freely available for a variety of extents, resolutions, and variables for both historical^25–28^ and future^29–31^ time periods.

Here we report on an effort to model and map the climate niches of more than 4000 forest insect and disease species in Canada, including high-profile invasive species that are already, or may soon become, established in the country^32^. This work makes use of several recently developed data sources to generate these niche models – and projects where suitable climate habitat may be found in the future. Importantly, we employ several species occurrence databases, including historical surveys coordinated by the Canadian Forest Service over the 1945–1995 period^33,34^. Historical and geographically extensive records such as these are critical to support species modeling efforts.

We further incorporate host distribution and abundance data to assess forest volume at risk by selected pest species under current and future climate. While such summaries do not offer the operational insights obtained by incorporating pest impacts into forest growth and mortality models^35,36^, they do provide rapid estimates of potential risk for a wide range of species. All outputs described here are available via a web application that allows users to search and download maps and models. This work is part of an ongoing effort to provide baseline information on the climate niches of plant and animal species across North America^37^.

## Methods

### Data sources

Georeferenced insect and disease occurrence data were obtained from several sources. The primary data source was the historical Forest Insect and Disease Survey (FIDS), which was jointly carried out by the Canadian Forest Service (CFS) and provincial resource management agencies across Canada over the 1945–1995 period^33,34^. For these surveys, forest technicians visited locations of reported pest activity and recorded all insect and disease species encountered. A second source comprised specimen vouchers from CFS regional centres and various collections, which have been incorporated into the Canadian Forest Invasive Alien Species (CanFIAS) database^34^. Together, these data sources accounted for some 403,296 records for 6,676 insect and disease species.

The occurrence data for these species were augmented with global occurrence records obtained in a series of downloads from the GBIF site^38–41^. Furthermore, occurrence records were obtained from GBIF for 10 species that were identified as species of special concern in the Canadian context^42^. These included alien invasive species that have arrived in Canada within the last 25 years (e.g., *Agrilus planipennis*) – or pose an imminent threat of arrival in Canada (e.g., *Bretziella fagacearum*) – and thus were not present in the original FIDS database. The GBIF data were screened using the following metadata filters: (1) presence of geospatial coordinates; (2) lack of geospatial issues; (3) year greater than 1950; and (4) record not based on a fossil specimen. Records for each species were further limited to those associated with a landmass and filtered at a 10-km resolution to reduce potential artefacts associated with duplicate records arising from intensive, localized survey efforts. Filters such as these have been identified as important tools to improve the quality and accuracy of GBIF data^43^. Only species with at least 30 occurrence records were considered for modelling and subsequent display on our web application. After applying these filters, we were left with some 3,393,954 observations (2,781,199 for insects; 612,755 for fungi ) for 4,009 species (3660 insects; 349 fungi).

Information regarding tree hosts was obtained from two sources. First, tree hosts associated with each insect and disease species were recorded during the FIDS surveys described above. These data were summarized to produce a list of known hosts associated with each pest species. Also, estimates of tree species distribution and abundance were obtained from national forest inventory grids at a 250 m resolution^23,24^. Together, these data sources allowed us to both identify potential tree hosts for a given insect/disease species and generate estimates of the area and volume of wood that may be at risk for a given time period and greenhouse gas emission scenario.

The following six climate variables were used for modelling species’ climate niches: mean annual temperature (MAT), mean of the daily minimum temperatures of the coldest month (MINTCM), mean of the daily maximum temperatures of the hottest month (MAXTHM), annual precipitation (PREC), precipitation of the coldest three month period (PRECCP), and precipitation of the hottest three month period (PRECHP). These variables have been shown to summarize important climate gradients (both mean and extreme conditions) for a variety of species^37^. Recent estimates of these variables were obtained by interrogating raster maps (i.e., grids) of global climate for the 1971–2000 period^27^ at each insect\disease occurrence location. The selection of this time period reflects the limited range of options available for global climate grids and coincides with the majority of the occurrence records in our database (67.9%).

Given the North American focus of this work, the resulting global models were projected onto Canada and the U.S. for current and future time periods using grids of climate for the six variables identified above. Grids of current climate (1971–2000) were generated by spatially interpolating U.S. and Canadian weather station data as described in previous work^25,28^. Future climate grids were generated using a delta approach^44^ to downscale general circulation model (GCM) outputs from the sixth coupled model intercomparison project (CMIP6) for 12 GCMs, three shared socioeconomic pathways (SSP1-2.6, SSP2-4.5, and SSP5-8.5)^45^, and three time periods (2011–2040, 2041–2070, and 2071–2100) as detailed in McKenney et al.^31^.

### Climate niche modelling

Two approaches were used for modelling forest insect and disease climate habitats. ANUCLIM^13^ – formerly Bioclim^12^ – is an early generation species distribution modelling software package that provides statistical summaries (i.e., min, max, mean, and percentiles) of climate at locations where a species is known to occur. This approach is known to have limitations due to the simple rectilinear nature of the resulting climate envelopes^46^, which tend to overestimate the actual range of the species in geographic space. However, we employed this method because it provides fundamental information on climate relationships in a simple and transparent manner and, as mentioned, provides conservative (i.e., inclusive) estimates of species’ climate niches.

MaxEnt is a machine learning method that estimates the distribution of a species by finding the distribution of “maximum entropy” (i.e., that is most spread out or closest to uniform), subject to a set of constraints defined by the environmental conditions at the occurrence locations^19,47^. This approach employs a regularization parameter (set to a value of 1 for the current work) which determines the smoothness of the resulting models and a variety of response functions (i.e., linear, product, quadratic, hinge, threshold, and categorical) to model potentially complex occurrence-environment relationships. An important consideration with MaxEnt models is the number and spatial extent of background points used in the analysis^48^. Given the global scope of the occurrence data and continental focus of the resulting models, we employed 10,000 random locations across the global landmass as background points for the current work. We recognize that customized background point selection may be a preferred approach, but given the large number of species and wide variation in spatial extent of occurrence data, we elected to use a global extent for this initial version of the work.

We employed these two modelling tools to address a range of study objectives. As noted, ANUCLIM is well-suited to identifying potential distributions; as such, it has been used to identify tentative range limits and potential search locations for rare and secretive species^49^. Alternatively, MaxEnt provides gridded estimates of occurrence probability, which allow insights into habitat suitability for a given species across a study area^19^. Given that species distributions are influenced by a myriad of biotic (e.g., competition) and abiotic (e.g., soils) factors that are not considered here, our outputs are best described as maps of potential distribution in relation to broad or meso-scale climate drivers^50^.

## Results and discussion

### Occurrence data

**Fig. 1:**
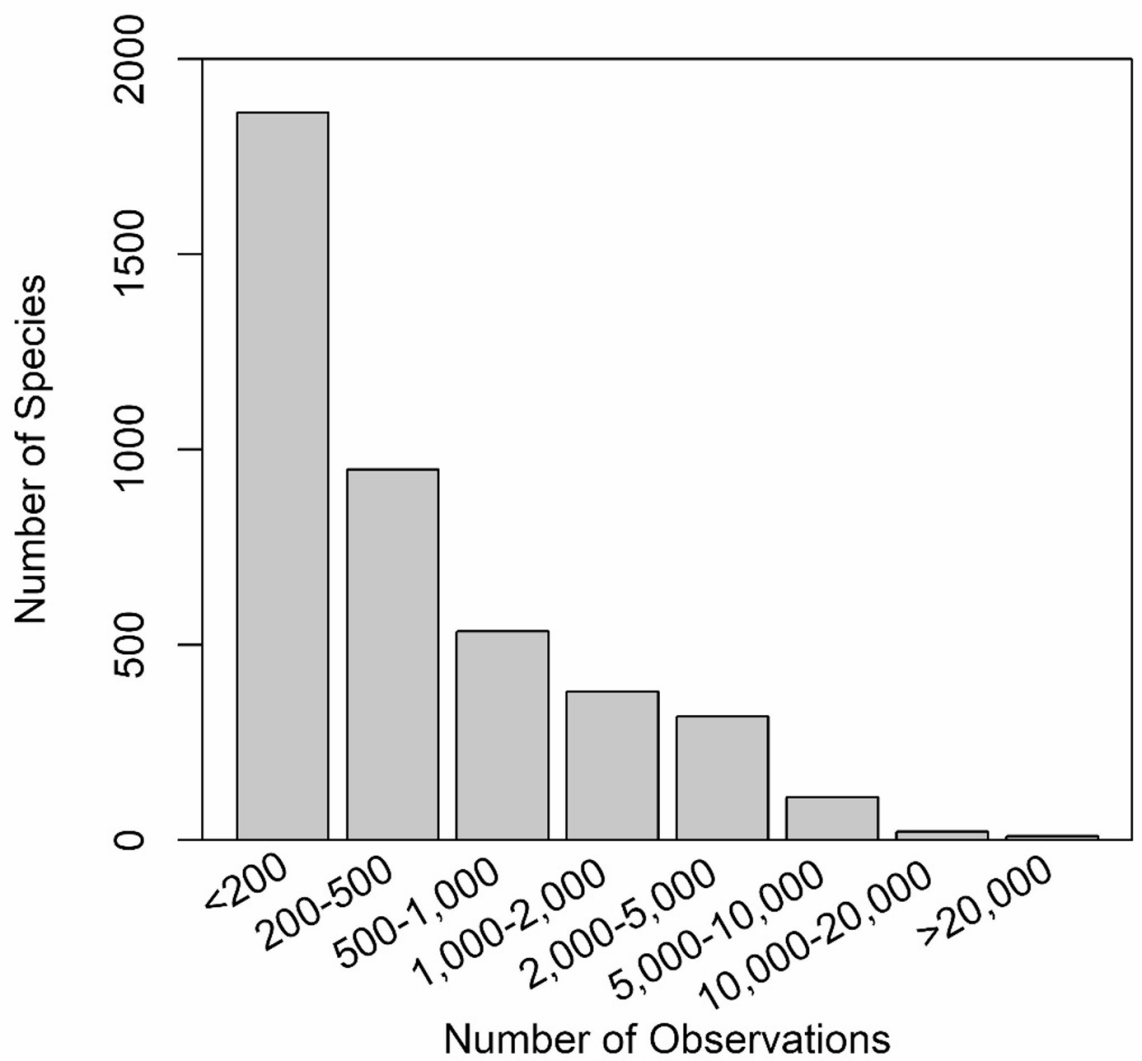
Number of species in each of eight frequency of occurrence classes from a database used to map forest insect and disease distributions in North America.

The median number of observations per species was 245, with values ranging from 30 to 48,554 (Fig. 1). The most common species in the dataset included various butterfly species (e.g., *Vanessa atalanta*; 48,554 observations), dragonflies (e.g., *Libellula quadrimaculata*; 20,563 observations), hornets and bees (e.g., *Vespa crabro*; 16,444 observations). Many of these species are not forest pests per se, but had a limited number of incidental observations in the original FIDS database, which were significantly augmented by GBIF data. We elected to include these species at the website as they may be of general scientific interest to users and, in rare cases, may develop into species of concern over time. Major forest insect pests represented in the dataset include: spongy moth (*Lymantria dispar*; 9747 observations), eastern spruce budworm (*Choristoneura fumiferana;* 9518 observations), forest tent caterpillar (*Malacosoma disstria;* 9275 observations), winter moth (*Operophtera brumata*; 7102 observations), and larch sawfly (*Pristiphora erichsonii*; 5,596). Important forest diseases include Armillaria root rot (*Armillaria mellea*; 8063 observations), Annosus root and butt rot (*Heterobasidion irregulare*; 4907 observations), Scleroderris canker (*Gremmeniella abietina*; 2095 observations), Dutch elm disease (*Ophiostoma ulmi*; 1,359 observations), and oak wilt (*Bretziella fagacearum*; 1133 observations).

**Fig. 2:**
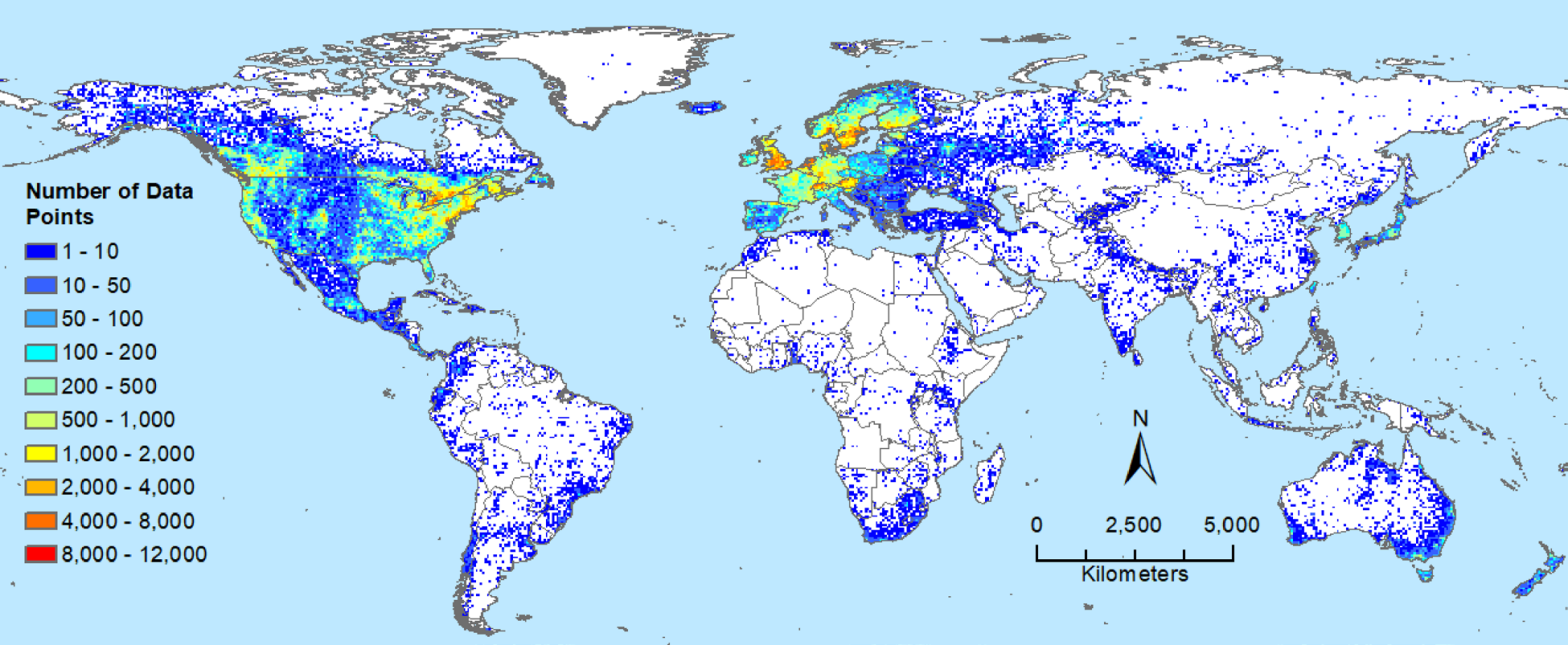
Number of forest insect and disease occurrence locations in each pixel (half-degree latitude and longitude) around the world. Occurrence locations were used to model species distributions in North America under current and future climate. Map produced using ArcGIS Pro^62^ and projected using EPSG:54,030.

Occurrence locations were spread over much of the forested area of Canada, with a preponderance of data points in the southern half of the country (Fig. 2). Locations outside Canada originated from the GBIF database and included major invasive threats, such as emerald ash borer (*Agrilus planipennis*) and Asian long-horned beetle (*Anoplophora glabripennis*). These global occurrence records were concentrated in the United States, Mexico, Europe, Japan, South Korea, southeastern Australia, and New Zealand (Fig. 2).

### Website and selected examples

A web application has been developed to allow public access to this work (https://cfs.cloud.nrcan.gc.ca/bmfid/). At the site, users can select a species of interest, as well as a time period, climate scenario, and modelling approach (ANUCLIM or MaxEnt). To facilitate rapid searching, pest species of high interest are highlighted on the front page of the web application. Species-specific model details, including accuracy estimates (e.g., AUC) and variable importance values are available at https://ftp.maps.canada.ca/pub/nrcan_rncan/Climate-archives_Archives-climatologiques/species_list/.

In the following paragraphs we present results for several species that demonstrate the value of the maps associated with this work. In selecting example species, we focused on those that are actively spreading within or near Canada and thus pose an ongoing or imminent threat. We intentionally avoid well-established, major Canadian forest pests – such as eastern spruce budworm (*Choristoneura fumiferana*) – for which advanced modelling efforts have already been undertaken.

**Fig. 3:**
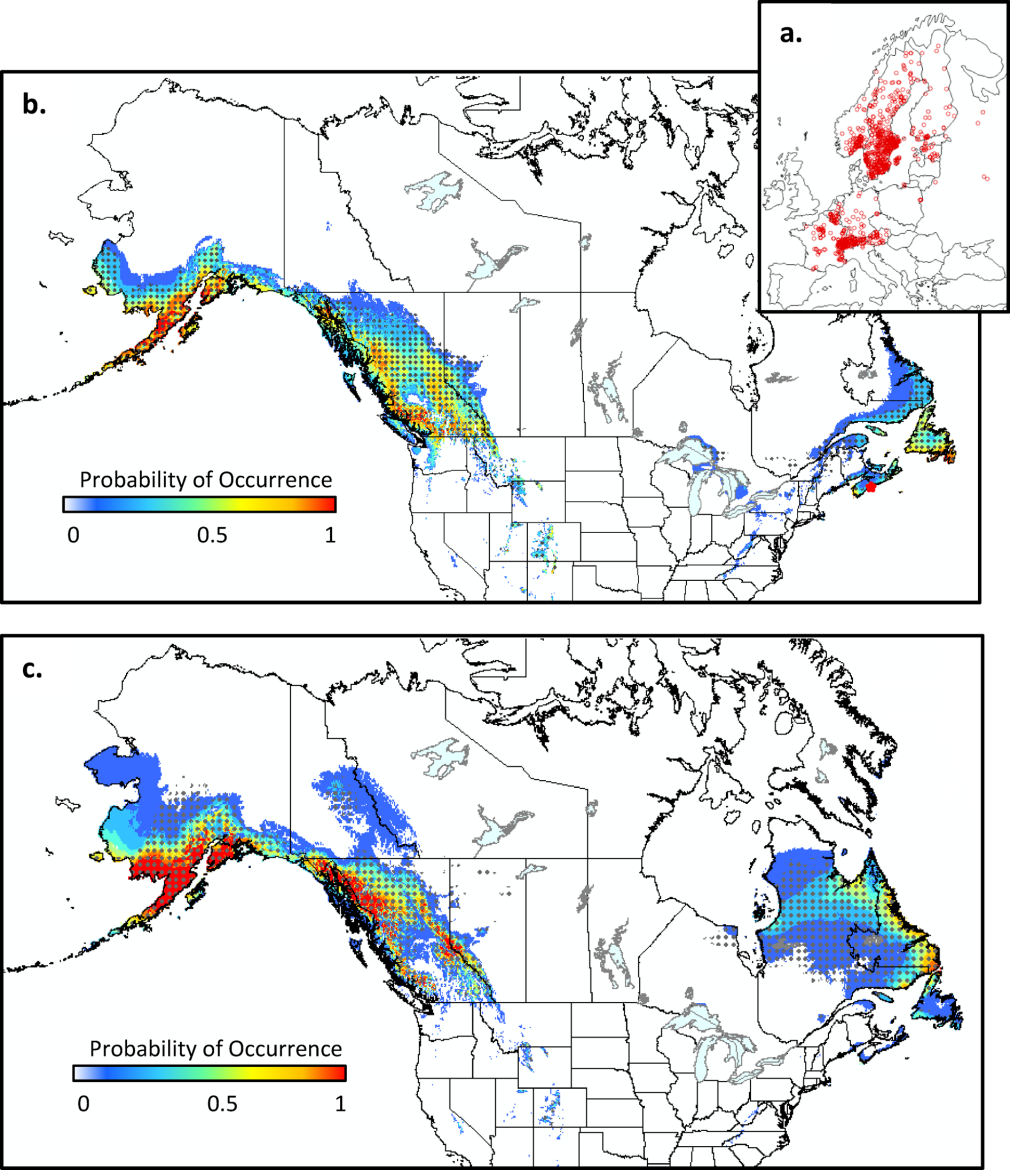
Climate suitability for brown spruce longhorned beetle (*Tetropium fuscum*) in North America based on occurrence data from its native range in Europe and Asia (a). Suitability is shown for the 1981–2010 period (b) and the 2041–2070 period under SSP2-4.5 (c) for both MaxEnt (colored contours) and ANUCLIM (dots) models. Maps produced using ArcGIS Pro^62^ and projected using EPSG:3978.

Brown spruce longhorn beetle (*Tetropium fuscum*) is native to Continental Europe and parts of Asia and infests a variety of spruce species (*Picea spp*). Global occurrence records for this species were obtained via GBIF download as described above (Fig. 3a). It was first recorded in North America at a park in Halifax, Nova Scotia in 1999^51^ - where it is thought to have been present for at least 10 years prior to discovery^52^. There are concerns that *T. fuscum* may spread across the Atlantic region and into the boreal forest where it could infest both black spruce (*Picea mariana*) and white spruce (*Picea glauca*), which are key ecological and economic tree species in this region. Our climate suitability maps (both MaxEnt and ANUCLIM) indicate that much of the suitable climate for this species is currently found in the eastern and western regions of the country (i.e., in the provinces of Nova Scotia, Prince Edward Island, Newfoundland and Labrador, far eastern Québec, and British Columbia; Fig. 3b). There are approximately 108.0 × 10^6^ m^3^ of host volume contained within this potentially suitable area in Canada. Suitable climate is projected to shift northward by the middle of the current century (Fig. 3c), but remain largely limited to the same provinces as those currently at risk. Under this projected climate niche, approximately 161.9 × 10^6^ m^3^ of host volume would be at risk in Canada, with much of the increase associated with low quality climate habitat opening up in far northern Québec. We note that climate suitability for this species is relatively low at the site of establishment in eastern Canada, which may, in part, explain the relatively slow spread of this organism since its discovery^52^.

**Fig. 4:**
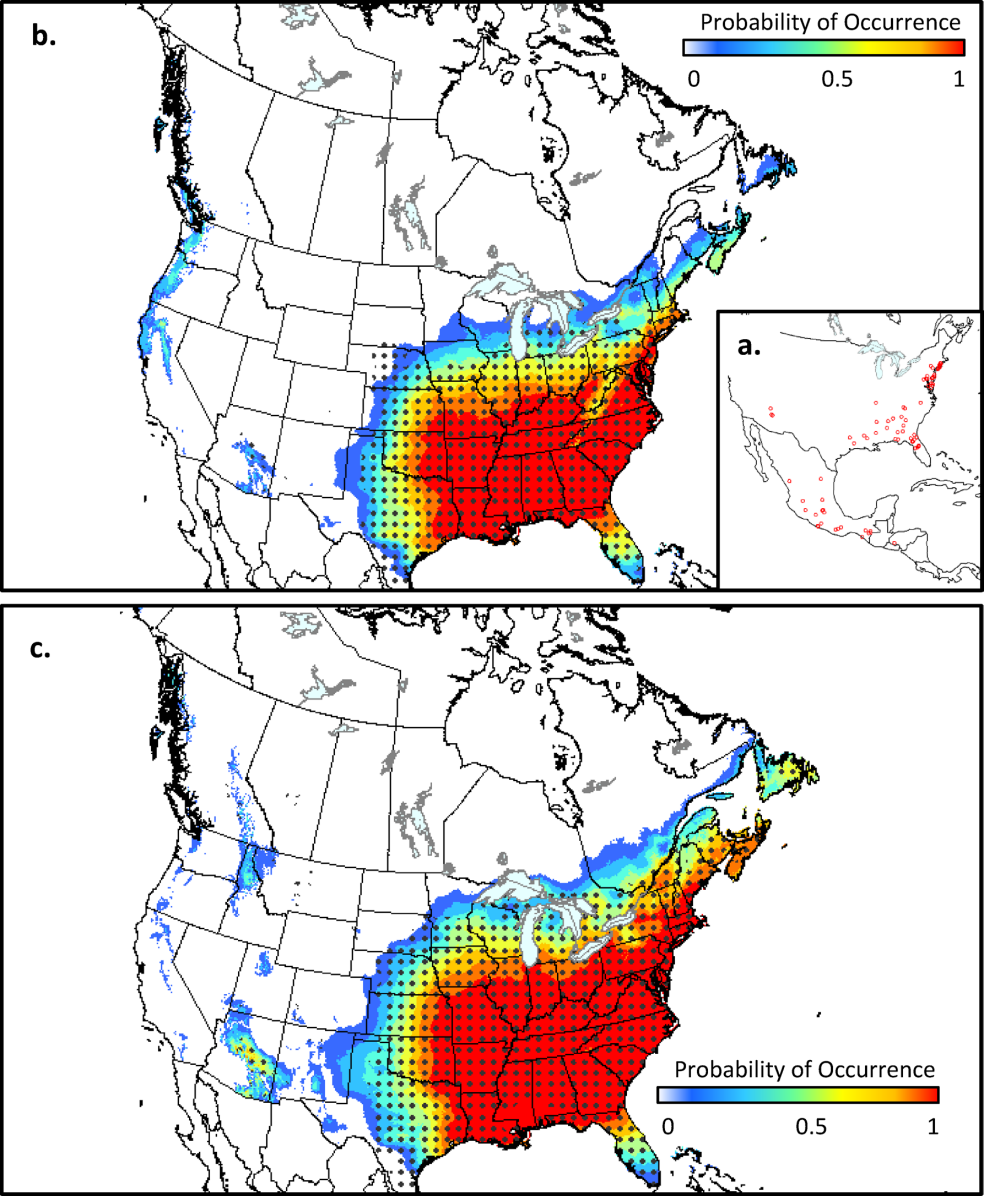
Climate suitability for southern pine beetle (*Dendroctonus frontalis*) in North America based on occurrence data from its native range in North and Central America (a). Suitability is shown for the 1981–2010 period (b) and the 2041–2070 period under SSP2-4.5 (c) for both MaxEnt (colored contours) and ANUCLIM (dots) models. Maps produced using ArcGIS Pro^62^ and projected using EPSG:3978.

Southern pine beetle (*Dendroctonus frontalis*) is a bark beetle, native to the southern United States, Mexico, and Central America (Fig. 4a). It infests various species of pine (*Pinus spp*) and is considered a significant forest pest across its native range^53^. There are concerns that it may infest forests in the northeasterrn U.S. and Canada under a warming climate^54,55^. Our climate niche maps indicate that there is currently low to moderate climate suitability for this species in southern Ontario, Québec, and portions of the Atlantic Provinces (Fig. 4b). However, by the middle of the current century, moderate to highly suitable climate conditions are projected for this same region, with less suitable conditions extending northward into central Ontario and Québec (Fig. 4c). These findings suggest that *D. frontalis* will likely move into southeastern Canada in the coming decades, where it could impact native pine species^54^; host volumes at risk in Canada are projected to increase from nearly zero under current climate to 0.8 × 10^6^ m^3^ by the middle of the current century under SSP2-4.5.

**Fig. 5:**
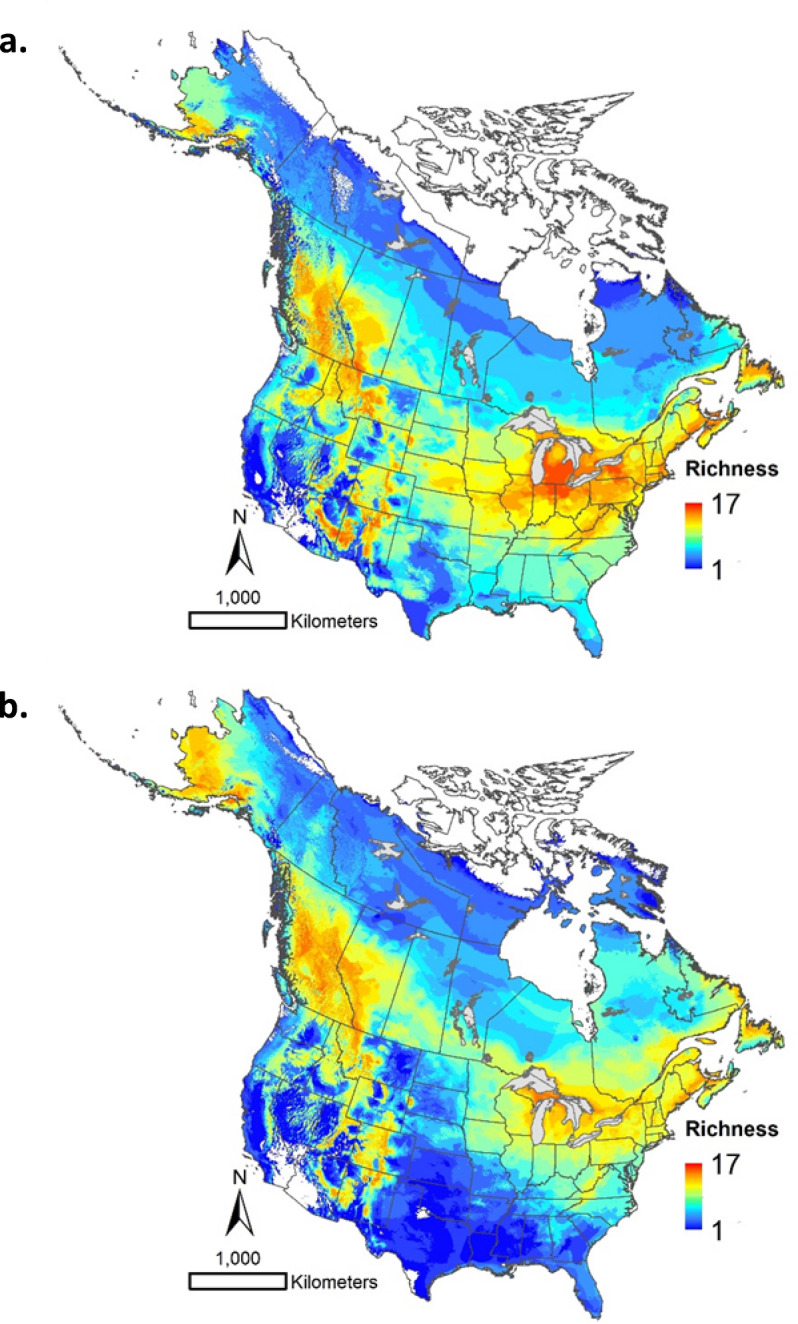
Climate envelope richness of North American bark beetles (genera *Dedroctonus*,* Ips*, and *Scolytus*) for (**a**) current (1971–2000) and (**b**) future (2041–2070) climate under a a composite of 12 general circulation models and a moderate emissions scenario (SSP2-4.5). See Figs. S1-S12 for individual GCM outputs. Maps produced using ArcGIS Pro^62^ and projected using EPSG:3978.

Our maps also allow users to explore shifts in species assemblages under climate change to elucidate macroscale patterns in potential pest hotspots. Here we overlay the climate envelope maps for 18 species of bark beetles in the genera *Dendroctonus*, *Ips*, and *Scolytus* (Table S1). These genera include some of the most damaging forest pests in North America and it is not clear how they may respond to rapid climate change over the coming decades. Under current climate, there are climate envelope hotspots in much of British Columbia, western Alberta, southern Alaska, and the mountain ranges of the western US; concentrations are also found in the Atlantic region of Canada, the northeastern US, and the Great Lakes region (Fig. 5a). By the middle of the current century, richness is projected to shift northward such that much of Canada has suitable climate for at least moderate levels of bark beetle richness (Fig. 5b) – a pattern that is relatively consistent across the 12 GCMs considered here (Fig. S1). Furthermore, bark beetle richness is projected to increase by five or more species in several key regions for industrial forestry across the country, including much of Québec, portions of northern Ontario, the prairie-forest ecotone in Manitoba and Saskatchewan, and much of northern Alberta and British Columbia (Fig. 5b). Though coarse, this analysis illustrates that northern forests may experience increased pest pressure as the century progresses. We note that, although overlay functionality is not currently available at the website, individual species maps can be obtained via download from the website – or by request to the corresponding author.

Our models also include high profile forest disease species, such as Dutch elm disease, Scleroderris canker, and butternut canker (*Ophiognomonia clavigignenti-juglandacearum*). Another species featured at the website is oak wilt (*Bretziella fagacearum*) – a significant disease of oak in the United States and recently discovered for the first time in Canada^56^(NAPPO 2023). In previous work, we presented current and future climate niche maps for this species to support an assessment of its potential economic impact in Canada, which included approximately CDN $0.5 billion in timber and street tree losses^57^(Pedlar et al. 2020). This study further illustrates the value of these maps for assessing current and future threats posed by expanding forest pests.

### Caveats, future Work, and concluding comments

There are several important caveats associated with the models and maps presented here. First, we recognize that the occurrence data may not provide a complete representation of the climatic preferences and constraints of some species. This, of course, is a concern for much of this type of research. Furthermore, the FIDS data was collected in response to reports of forest insect/disease activity and thus is biased both toward high profile species with obvious impacts and toward the southern half of the country where the majority of survey crews and human infrastructure such as roads were located. Similar biases and inaccuracies have also been recognized as a concern with the GBIF data^58^. One drawback with providing climate niche maps for many insect and disease species is that it necessarily limits the extent to which models can be tailored to any given species. Consequently, we recognize and stress that this effort is necessarily a work in progress; however, we believe there is value in providing potential users and the forestry community with these preliminary findings and updating the maps and models as more data becomes available. Indeed, efforts are underway to incorporate these products into annual harvest level estimates in the province of Québec, Canada^59^.

There are several near-term upgrades planned for the website. These include providing users with more details on model outputs, such as estimates of model accuracy (e.g., AUC and TSS statistics) and measures of importance for the various climate variables included in the analysis. In the meantime, users can access model details at https://ftp.maps.canada.ca/pub/nrcan_rncan/Climate-archives_Archives-climatologiques/species_list/. Ongoing efforts will also involve regular updating of the GBIF occurrence data, including ongoing quality control improvements via the various metadata filters available at the GBIF site^43^. Finally, we are exploring the possibility of incorporating algorithms that would facilitate automated, species-specific tuning of model parameters^60,61^, thus allowing customized MaxEnt models for the large pool of species considered here.

This work provides an unprecedented compilation of baseline information on the climate niches of forest insect and disease species in Canada. Our effort made use of a significant quantity of data from historical CFS surveys, GBIF, and various other data sources. All information has been made publically available via a web application which allows the climate niche models and maps to be explored and downloaded. This product is intended as a tool to allow forest managers to better understand how these species may respond to projected climate change over the course of the current century.

## Supplementary Information

Below is the link to the electronic supplementary material.

Supplementary Material 1

## Data Availability

Maps for all species of forest insects and diseases included in this study are available at: (https:/‌cfs.cloud.nrcan.gc.ca/‌bmfid‌).
