# Supplementary material for: Mapping the climate niches of forest insects and diseases in Canada under current and future climate
Source: Sci Rep. 2025 Nov 20;15:40996. doi: 10.1038/s41598-025-24833-8 (PMC12635192; doi:10.1038/s41598-025-24833-8)
Supplement: Supplementary file 1 — Supplementary Material 1 [file 41598_2025_24833_MOESM1_ESM.docx]

**Supplementary Information**

Mapping Forest Insect and Disease Distributions in Canada under Current and Future Climate


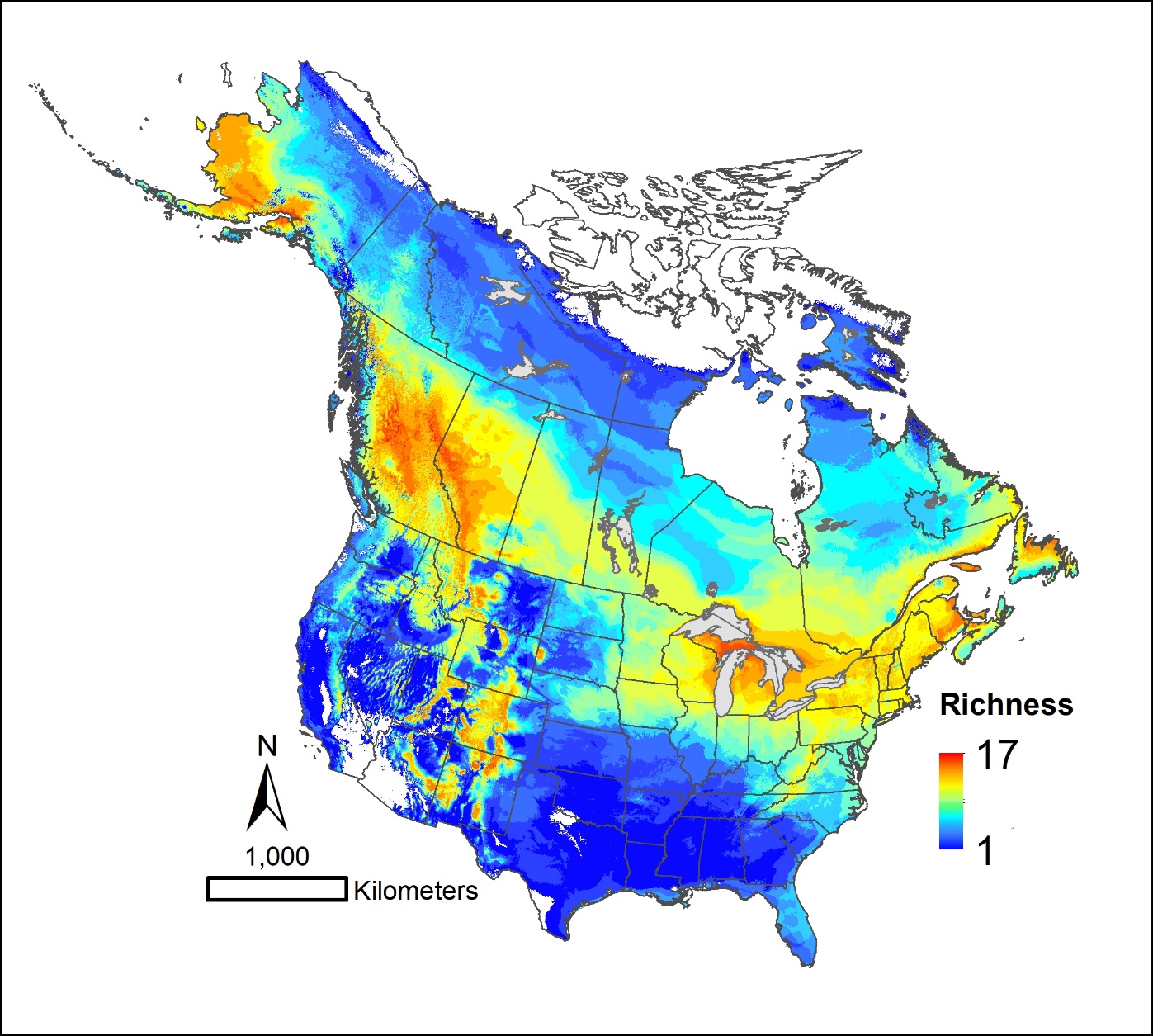


**Figure S1**. Species richness of bark beetles in North America – projected for the 2041-2070 period under the BCC-CSM2-MR climate model and the SSP2-4.5 emission scenario. Map produced using ArcGIS Pro Version 3.0 (<https://www.esri.com/en-us/arcgis/products/arcgis-pro/overview>).


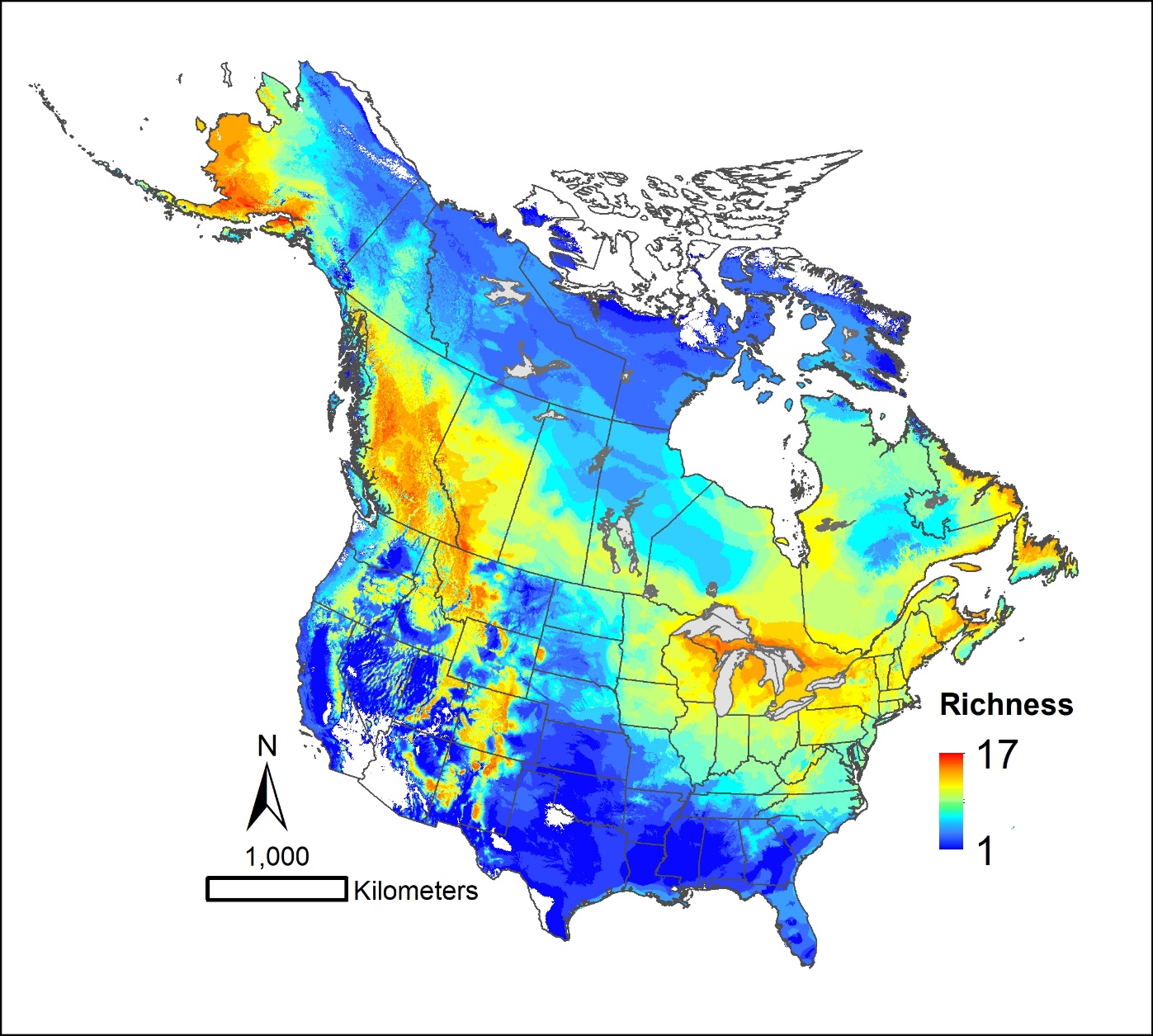


**Figure S2**. Species richness of bark beetles in North America – projected for the 2041-2070 period under the CMCC-ESM2 climate model and the SSP2-4.5 emission scenario. Map produced using ArcGIS Pro Version 3.0 (<https://www.esri.com/en-us/arcgis/products/arcgis-pro/overview>).


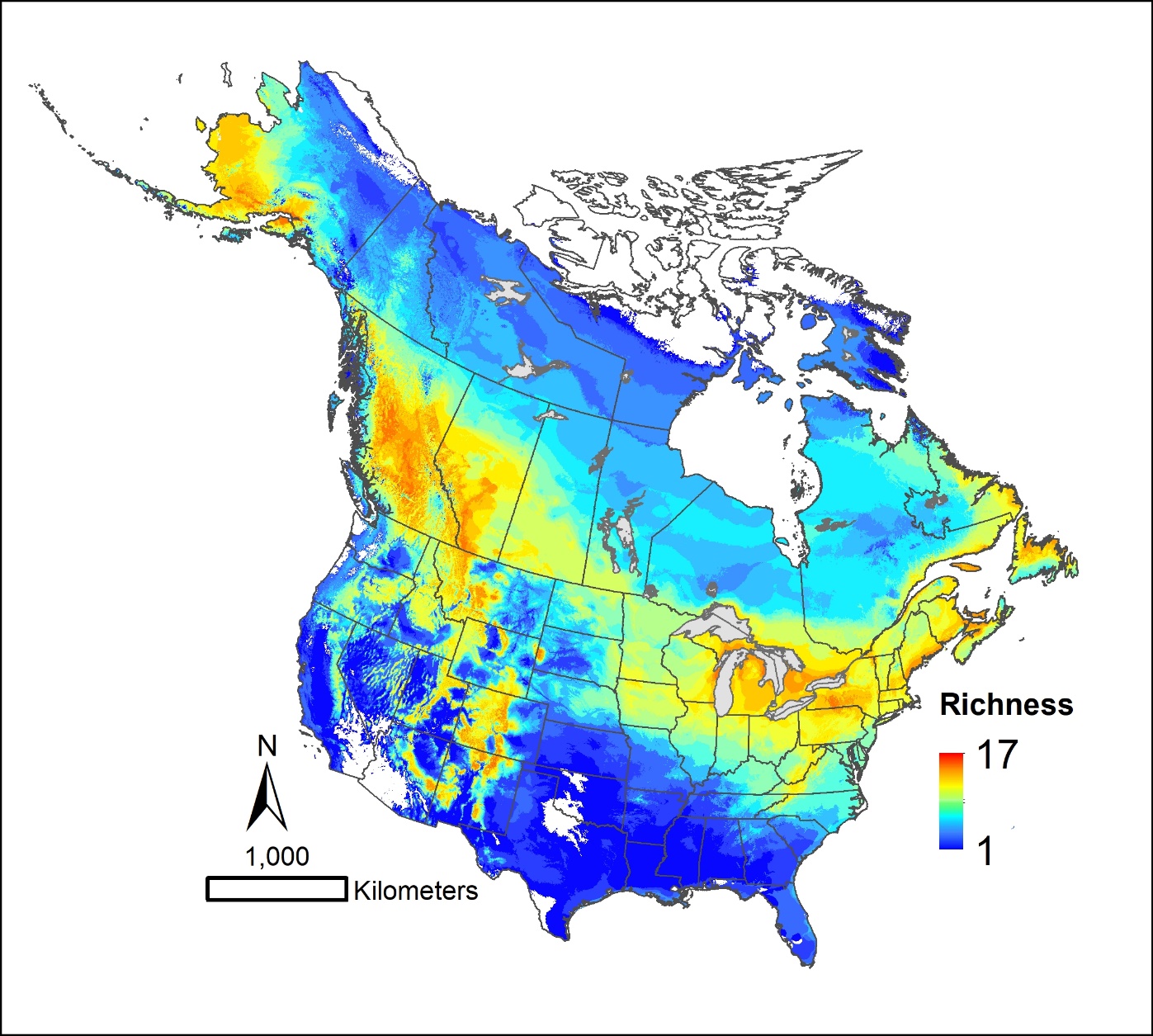


**Figure S3**. Species richness of bark beetles in North America – projected for the 2041-2070 period under the EC-Earth3-Veg climate model and the SSP2-4.5 emission scenario. Map produced using ArcGIS Pro Version 3.0 (<https://www.esri.com/en-us/arcgis/products/arcgis-pro/overview>).

**
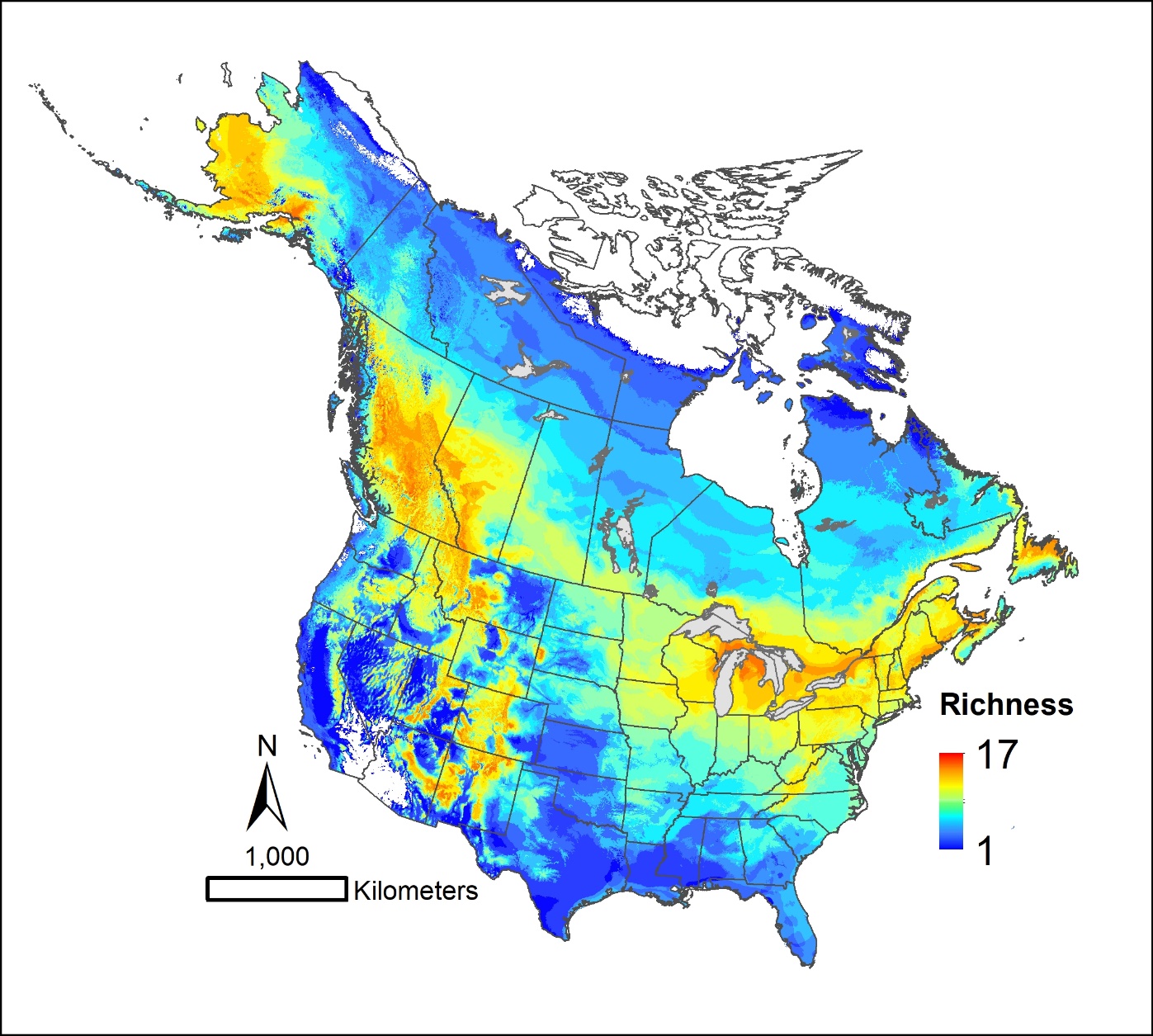
**

**Figure S4**. Species richness of bark beetles in North America – projected for the 2041-2070 period using the FGOALS-g3 climate model and the SSP2-4.5 emission scenario. Map produced using ArcGIS Pro Version 3.0 (<https://www.esri.com/en-us/arcgis/products/arcgis-pro/overview>).


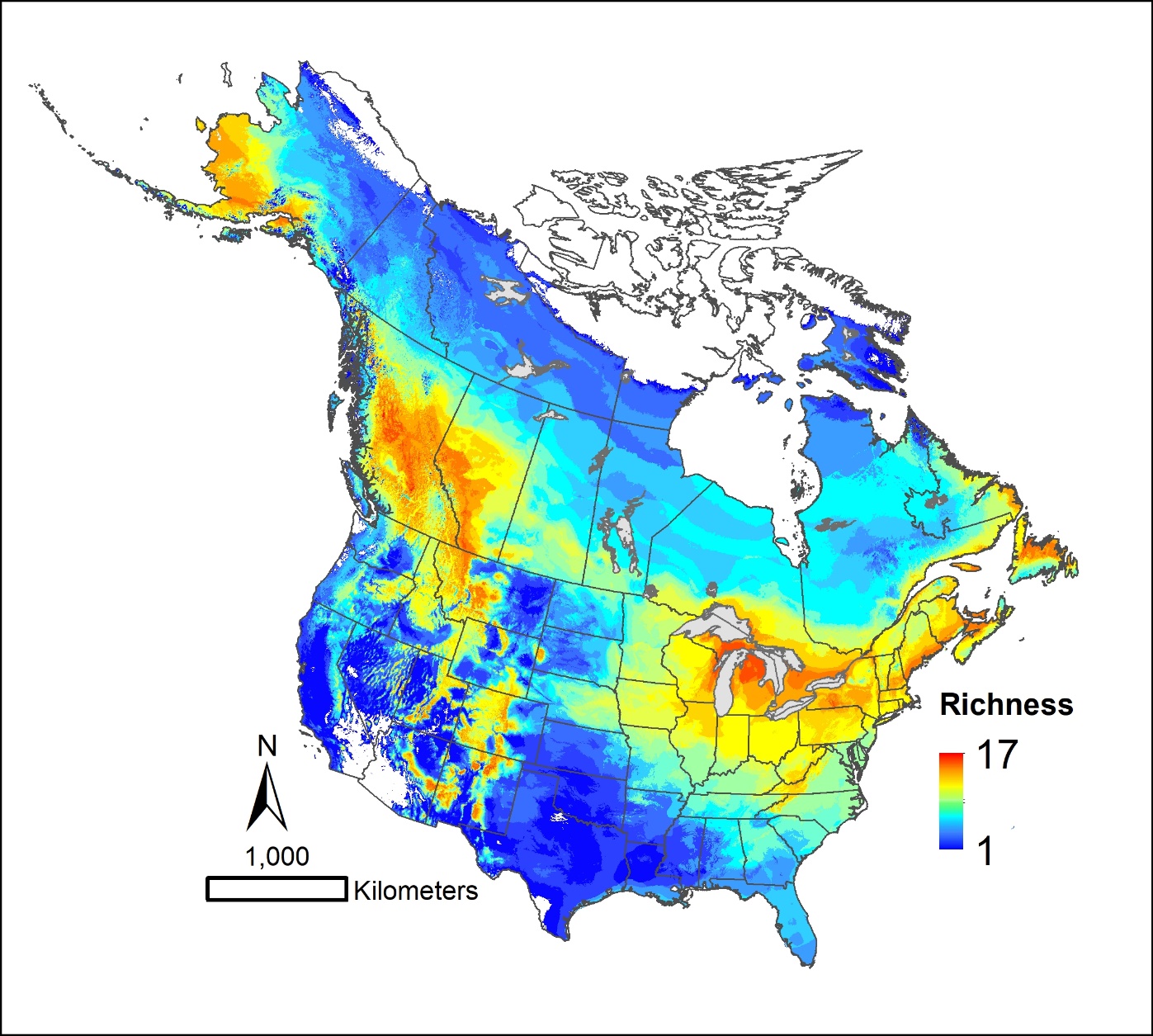


**Figure S5**. Species richness of bark beetles in North America – projected for the 2041-2070 period using the INM-CM5-0 climate model and the SSP2-4.5 emission scenario. Map produced using ArcGIS Pro Version 3.0 (<https://www.esri.com/en-us/arcgis/products/arcgis-pro/overview>).


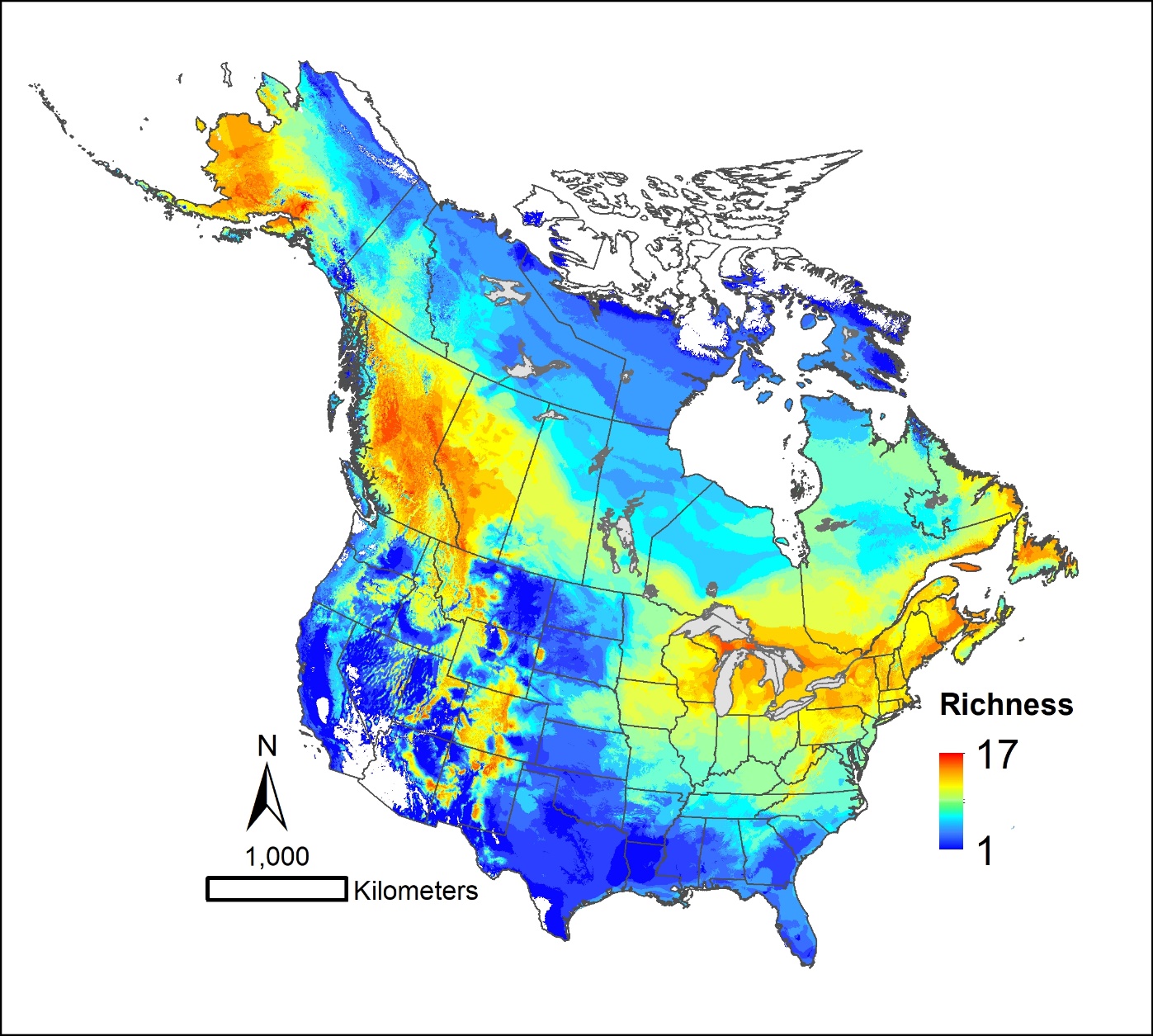


**Figure S6**. Species richness of bark beetles in North America – projected for the 2041-2070 period using the IPSL-CM6A-LR climate model and the SSP2-4.5 emission scenario. Map produced using ArcGIS Pro Version 3.0 (<https://www.esri.com/en-us/arcgis/products/arcgis-pro/overview>).


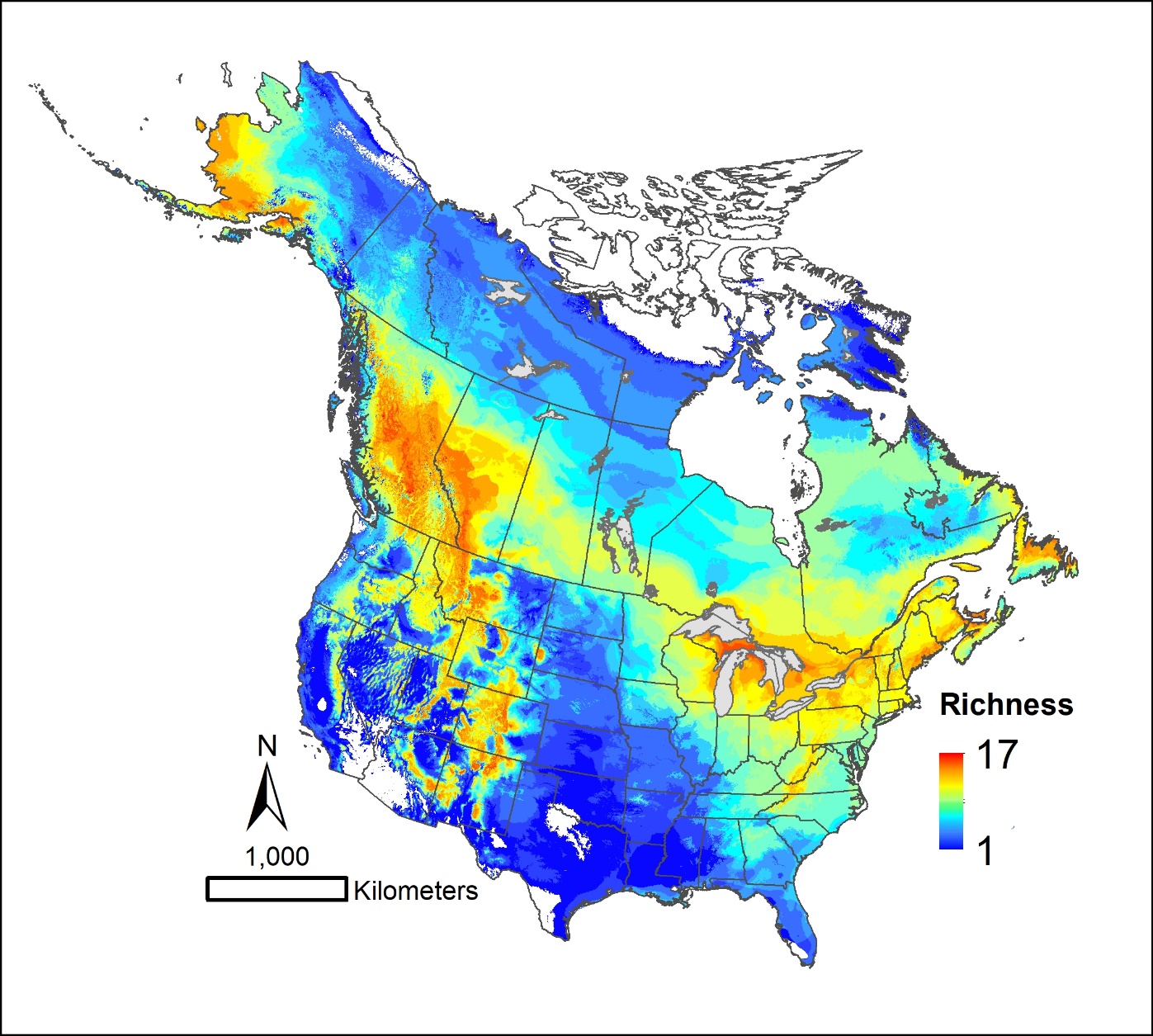


**Figure S7**. Species richness of bark beetles in North America – projected for the 2041-2070 period using the MIROC-ES2L climate model and the SSP2-4.5 emission scenario. Map produced using ArcGIS Pro Version 3.0 (<https://www.esri.com/en-us/arcgis/products/arcgis-pro/overview>).


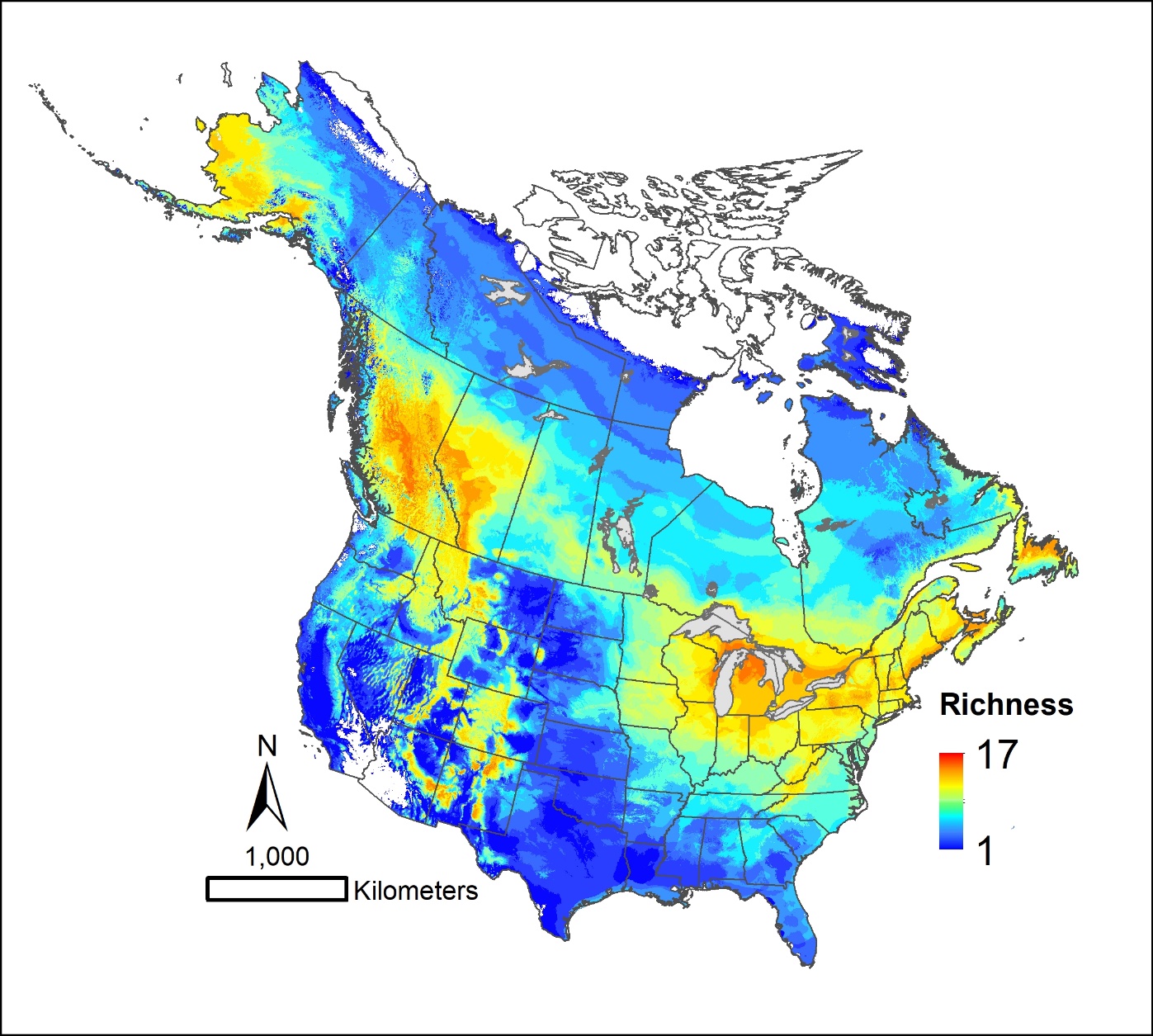


**Figure S8**. Species richness of bark beetles in North America – projected for the 2041-2070 period using the MPI-ESM1-2-HR climate model and the SSP2-4.5 emission scenario. Map produced using ArcGIS Pro Version 3.0 (<https://www.esri.com/en-us/arcgis/products/arcgis-pro/overview>).

**
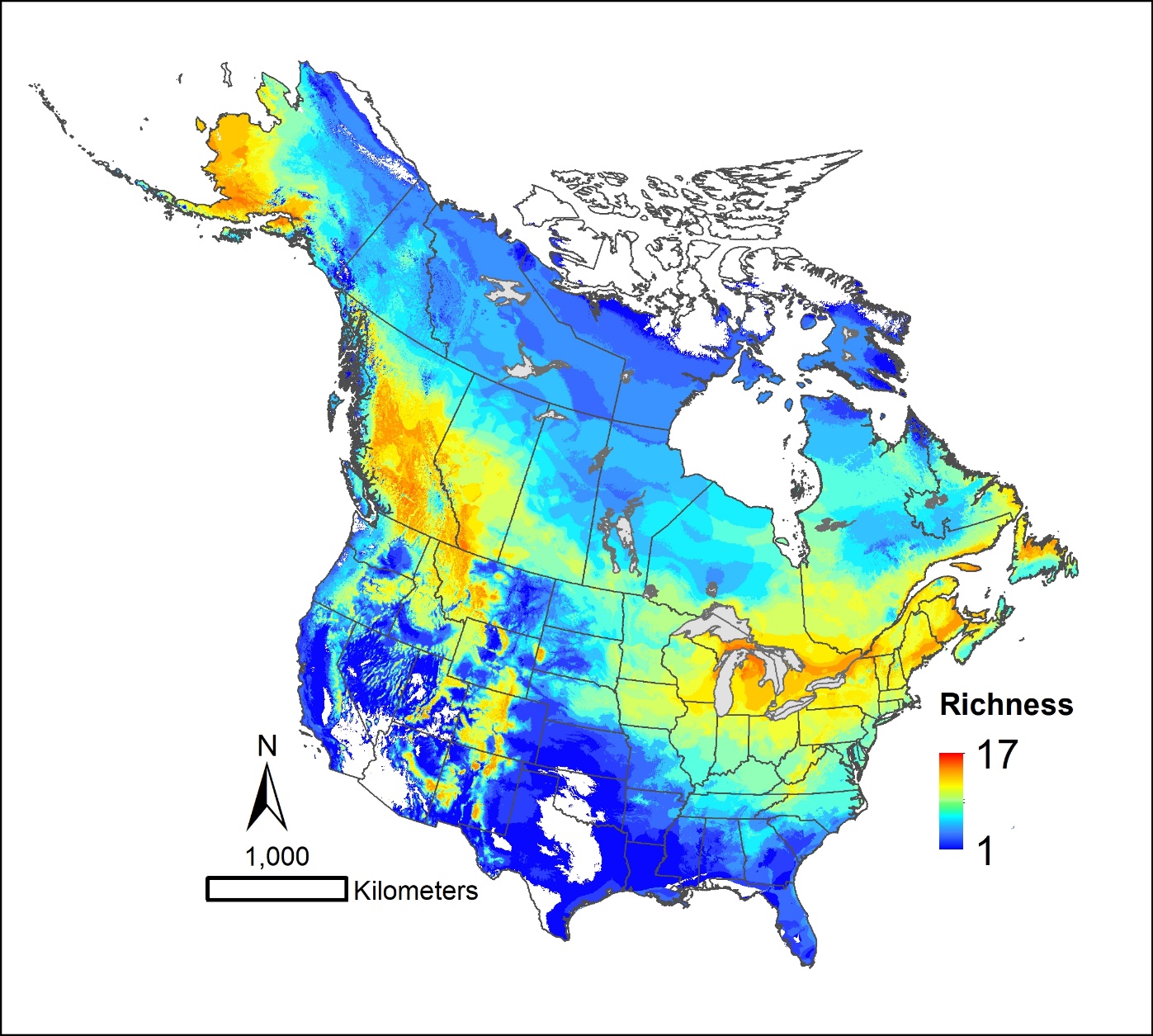
**

**Figure S9**. Species richness of bark beetles in North America – projected for the 2041-2070 period using the MRI-ESM2-0 climate model and the SSP2-4.5 emission scenario. Map produced using ArcGIS Pro Version 3.0 (<https://www.esri.com/en-us/arcgis/products/arcgis-pro/overview>).


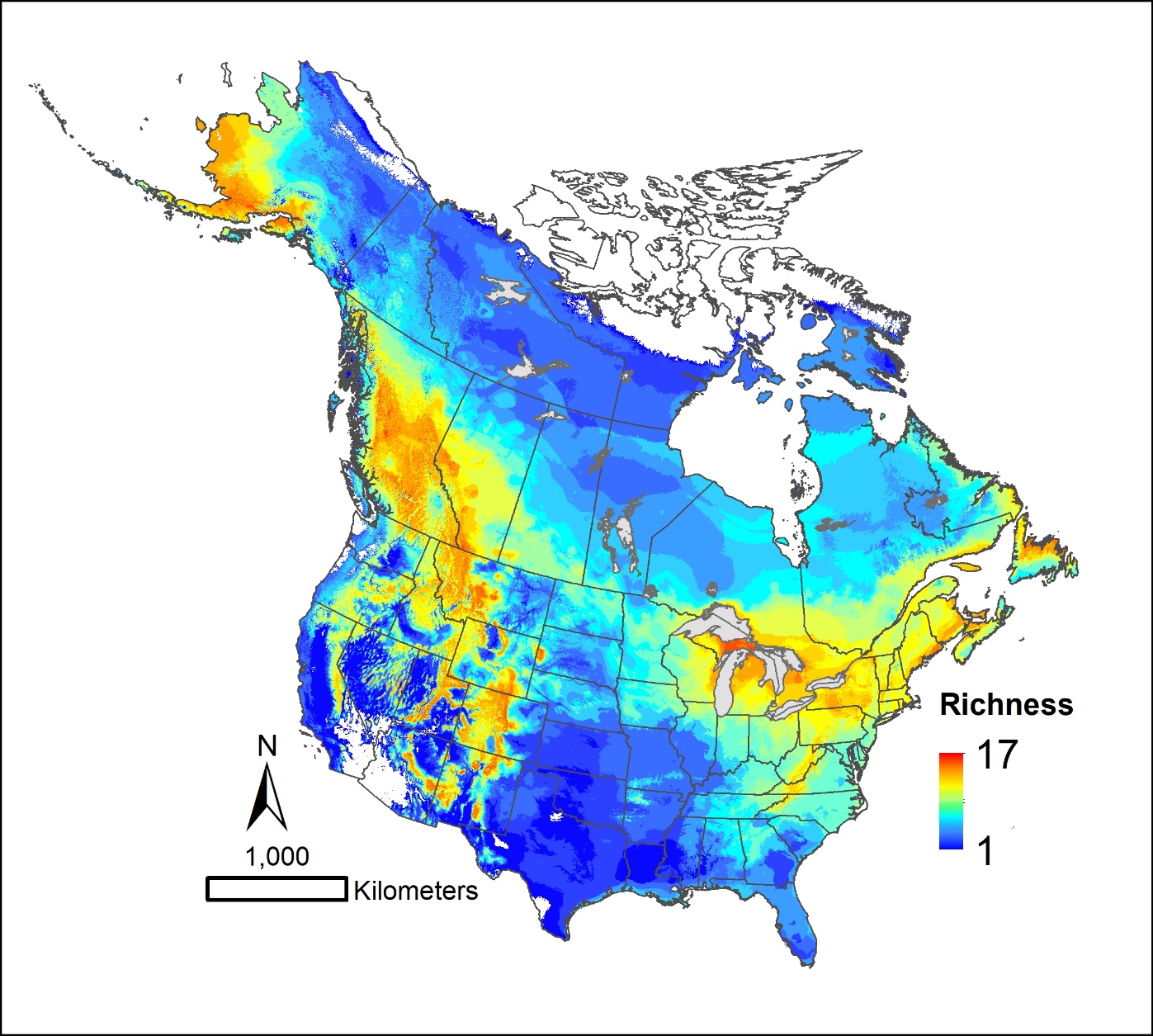


**Figure S10**. Species richness of bark beetles in North America – projected for the 2041-2070 period using the NorESM2-LM climate model and the SSP2-4.5 emission scenario. Map produced using ArcGIS Pro Version 3.0 (<https://www.esri.com/en-us/arcgis/products/arcgis-pro/overview>).

**
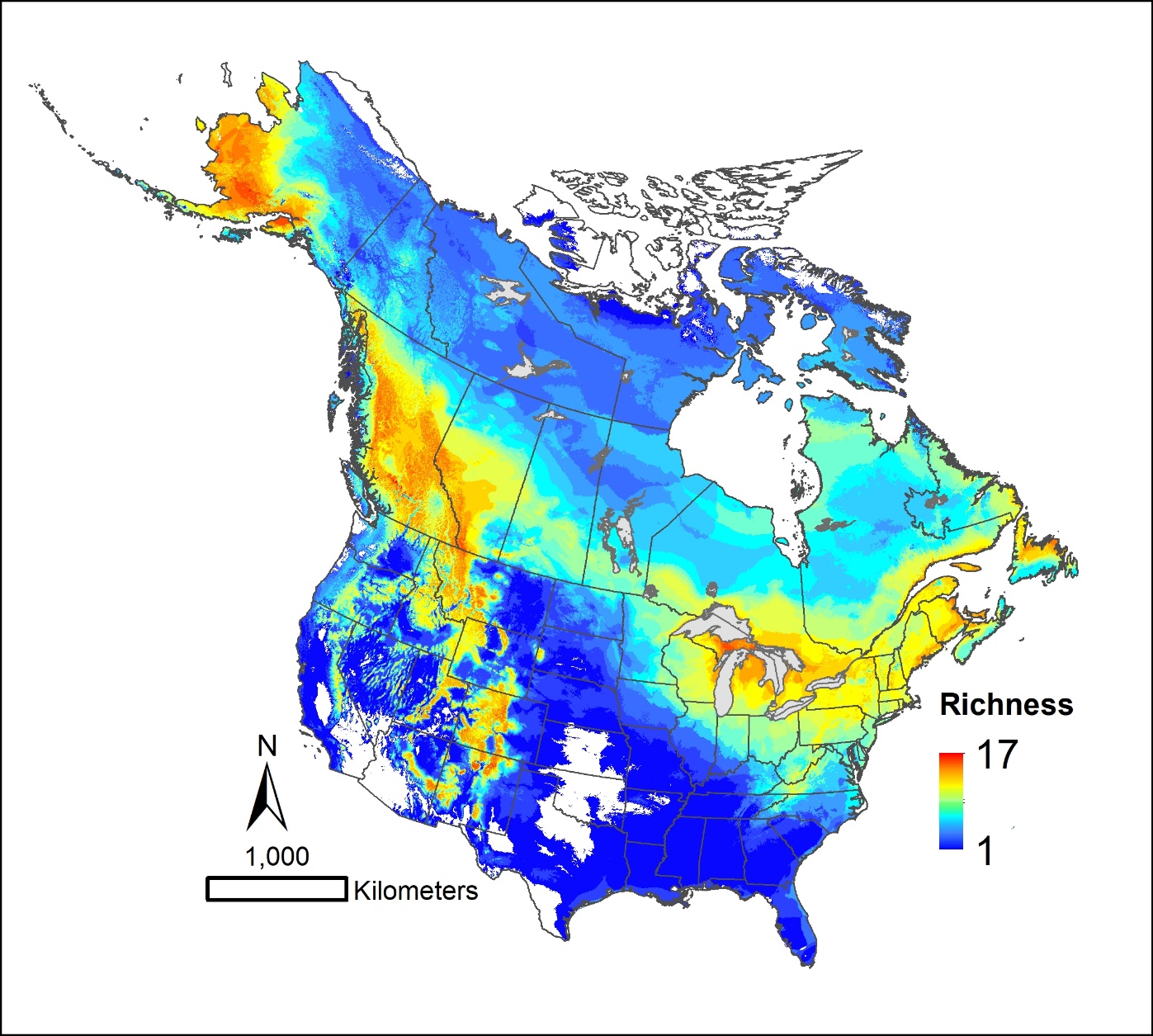
**

**Figure S11**. Species richness of bark beetles in North America – projected for the 2041-2070 period using the TaiESM1 climate model and the SSP2-4.5 emission scenario. Map produced using ArcGIS Pro Version 3.0 (<https://www.esri.com/en-us/arcgis/products/arcgis-pro/overview>).


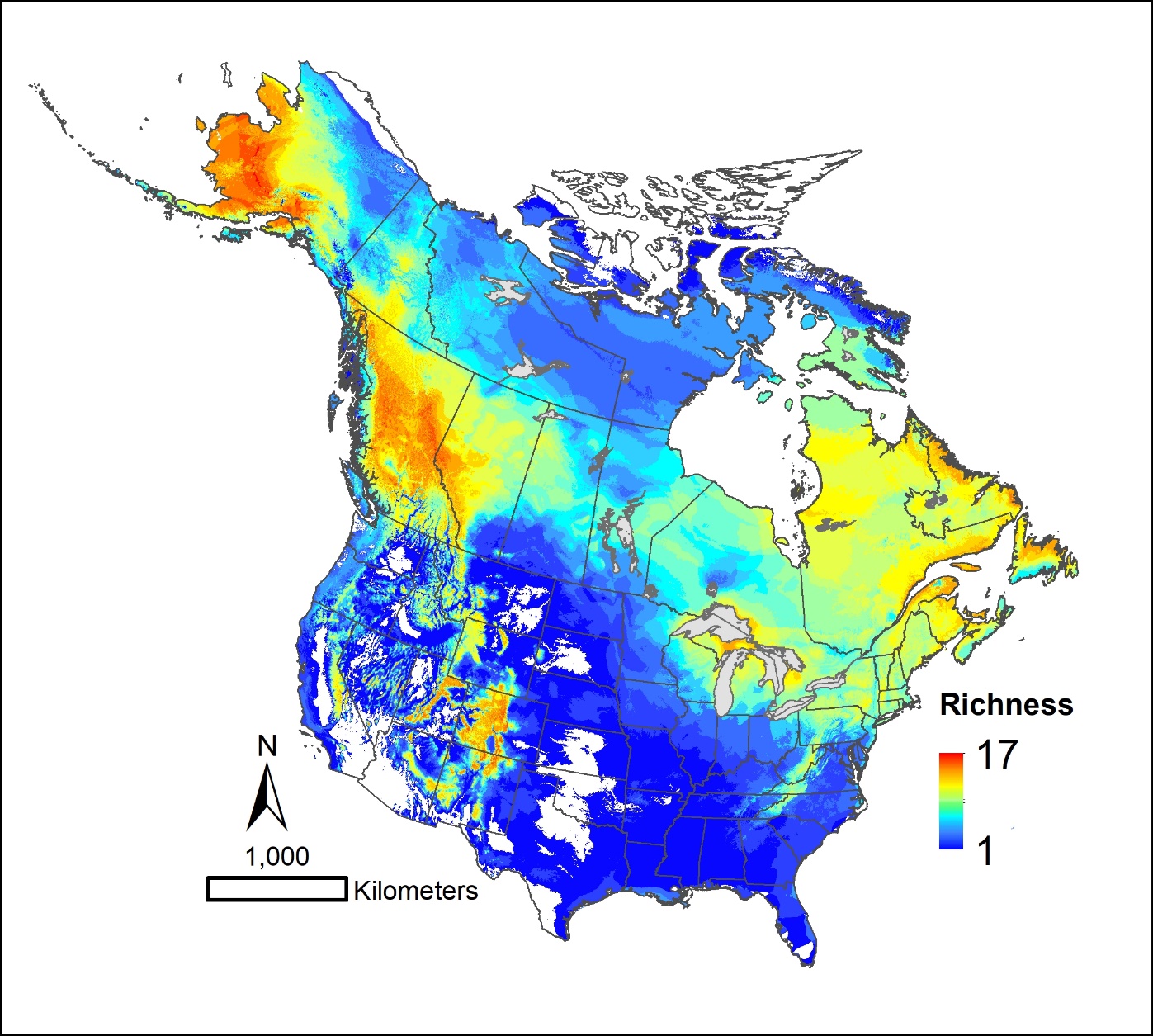


**Figure S12**. Species richness of bark beetles in North America – projected for the 2041-2070 period using the UKESM1-0-LL climate model and the SSP2-4.5 emission scenario. Map produced using ArcGIS Pro Version 3.0 (<https://www.esri.com/en-us/arcgis/products/arcgis-pro/overview>).

| Species | Common name | Host notes | Distribution notes |
| --- | --- | --- | --- |
| Dendroctonus brevicomis | western pine beetle | Primarily ponderosa pine (*Pinus pondersa*) | Native to southern B.C., western U.S. |
| Dendroctonus frontalis | southern pine beetle | Many pine species in eastern U.S. | Native to southern U.S., Mexico, and Central America |
| Dendroctonus ponderosae | mountain pine beetle | At least 8 species of pine in western N.A. | Native to western North America from Mexico to central British Columbia |
| Dendroctonus pseudotsugae | Douglas-fir beetle | Primarly Douglas-fir (*Pseudotsuga menziesii*) | Native to western North America |
| Dendroctonus rufipennis | spruce beetle | Various spruces, including white (*Picea glauca*), Englemann (*Picea engelmannii*), sitka (*Picea sitchensis*), and blue spruce (*Picea pungens*) | Native to northern North America |
| Dendroctonus simplex | eastern larch beetle | Primarily eastern tamarack (*Larix laricina*) and western tamarack (*Larix occidentalis*) | Native to northern North America |
| Dendroctonus valens | red turpentine beetle | White fir (*Abies concolor*) and various species of spruce (*Picea*) and pine (*Pinus*) | Native to Cental America, Mexico, U.S., and southern Canada |
| Ips calligraphus | six-spined ips | Many pine species in eastern U.S. | Primarily eastern U.S. and Mexico |
| Ips grandicollis | eastern five-spined engraver | Many species of pine and larch | Eastern Canada and U.S., Mexico, and Central America |
| Ips integer | Ips integer | Various pine species | Primarly Mexico and western U.S. |
| Ips mexicanus | Monterey pine engraver | Various pine species | North and Central America |
| Ips perturbatus | northern spruce engraver | Various spruce species | Northern U.S. and Canada |
| Ips pini | pine engraver | Various pine species | Northern Canada and Alaska to northern Mexico |
| Ips tridens | three-toothed bark weevil | Various spruce species | Native to western North America |
| Scolytus mali | larger shothole borer | Broad-leaved species; particularly fruit trees | Native to Europe; introduced to North America |
| Scolytus multistriatus | European elm bark beetle | Broad-leaved species; particularly elm | Native to Europe; introduced to North America |
| Scolytus piceae | phloem bark weevil | Various spruce species | Northern North America |
| Scolytus rugulosus | shothole borer | Broad-leaved species; particularly fruit trees | Native to Europe; introduced to North America |

Table S1. Species used to assess potential bark beetle shifts under climate change.
